# Fine-scale malaria risk mapping from routine aggregated case data

**DOI:** 10.1186/1475-2875-13-421

**Published:** 2014-11-03

**Authors:** Hugh JW Sturrock, Justin M Cohen, Petr Keil, Andrew J Tatem, Arnaud Le Menach, Nyasatu E Ntshalintshali, Michelle S Hsiang, Roland D Gosling

**Affiliations:** Global Health Group, University of California, San Francisco, SF USA; Clinton Health Access Initiative, Boston, MA USA; Department of Ecology and Evolutionary Biology, Yale University, New Haven, USA; Department of Geography and Environment, University of Southampton, Southampton, UK; Fogarty International Center, National Institutes of Health, Bethesda, USA; Flowminder Foundation, Stockholm, Sweden; Department of Pediatrics, University of Texas Southwestern Medical Center, Dallas, TX USA

## Abstract

**Background:**

Mapping malaria risk is an integral component of efficient resource allocation. Routine health facility data are convenient to collect, but without information on the locations at which transmission occurred, their utility for predicting variation in risk at a sub-catchment level is presently unclear.

**Methods:**

Using routinely collected health facility level case data in Swaziland between 2011–2013, and fine scale environmental and ecological variables, this study explores the use of a hierarchical Bayesian modelling framework for downscaling risk maps from health facility catchment level to a fine scale (1 km x 1 km). Fine scale predictions were validated using known household locations of cases and a random sample of points to act as pseudo-controls.

**Results:**

Results show that fine-scale predictions were able to discriminate between cases and pseudo-controls with an AUC value of 0.84. When scaled up to catchment level, predicted numbers of cases per health facility showed broad correspondence with observed numbers of cases with little bias, with 84 of the 101 health facilities with zero cases correctly predicted as having zero cases.

**Conclusions:**

This method holds promise for helping countries in pre-elimination and elimination stages use health facility level data to produce accurate risk maps at finer scales. Further validation in other transmission settings and an evaluation of the operational value of the approach is necessary.

**Electronic supplementary material:**

The online version of this article (doi:10.1186/1475-2875-13-421) contains supplementary material, which is available to authorized users.

## Background

Mapping malaria risk is an integral component of effective and efficient resource allocation. Traditionally, risk maps have been based on infection prevalence data collected during cross-sectional surveys such as MIS (malaria indicator surveys) or national school surveys [1, 2]. In low transmission settings, because there are so few infections possible to detect in the community, the sample size requirements for both estimation and spatial prediction of infection prevalence are very large making such surveys operationally and financially unfeasible [3, 4]. Furthermore, such surveys are typically conducted every few years, yet elimination programs require timely, if not real-time, information on malaria transmission to ensure they can respond rapidly to changing epidemiological circumstances.

Using data from Swaziland, Cohen *et al*. [5] showed that routine surveillance data can be used to generate fine scale risk maps (relative probability of being an incident case) if the household location of cases is known using ecological niche modelling. Georeferenced case data is available in Swaziland due to a strong active surveillance programme, which aims to follow up and map all cases to their home [5, 6]. In most low transmission settings, however, household locations of cases is often not known, with routine malaria surveillance systems reporting only the numbers of cases confirmed at each health facility. Typically, this data is aggregated to sub-district or district level before modelling, making predictions only possible at this coarse scale [7, 8]. A method to predict fine-scale transmission hotspots to village and sub-village level from health facility level would give programmes actionable information to target interventions without having to first geolocate cases back to household.

Cross-scale prediction - predicting at fine-scales using data available at coarser scales - is a familiar issue encountered by ecology researchers [9–11]. One approach, tested by several researchers is 'direct prediction’ whereby data are modelled at coarse scale and inferred statistical relationships are then projected onto the fine-scale covariates to produce fine-scale predictions [9, 11]. Modelling data at one scale and predicting at another can, however, lead to spurious and unreliable predictions. An alternative approach is termed 'point sampling’ whereby a defined number of randomly selected pixels within each coarse scale unit are selected and modelled with the outcome [9, 12]. While this avoids some of the drawbacks of direct prediction, the arbitrary nature of selecting pixels within coarse units may lead to unreliable predictions.

More recently, Keil *et al*. applied a Hierarchical Bayesian Modelling (HBM) approach which treated fine-scale presences/absences of bird species in California as latent, or unobserved, variables, which were modelled as a function of observed fine-grain environmental variables and constrained by observed coarse-grain presences/absences using logistic regression [13]. This approach allows fitting of realistic and multi-scale spatial models with both the observation and the process components, and also enables estimation of the uncertainty in the fine scale predictions. Here, similar modelling methods were applied to predict malaria risk at fine spatial resolution from routine health facility level case data in Swaziland with the goal of devising methods that can be applied to other settings where aggregate health facility data are routinely reported. As health facility case data are influenced by treatment-seeking behaviour of the population, information on treatment-seeking, collected during the most recent Swaziland MIS, are incorporated into the modelling process.

## Methods

### Case data

Data were extracted from the Swaziland Malaria Surveillance Database System, which includes information on the number of RDT and microscopy confirmed malaria cases identified at each of the 165 georeferenced public health facilities that offer malaria diagnosis, as well as active case investigation of all confirmed malaria cases contacted and followed-up. Active case investigation data includes the GPS location of case households and information on travel history from the four weeks prior to permit classification of whether infections were locally acquired or imported. As there appear to be different drivers of transmission between seasons [14], for the purpose of this study only local cases presenting during high season (January 1 to April 30) between 2011–2013 that received an investigation were included in the analyses. A case is classified as local if the case resides in, or travels to, a receptive area of Swaziland (determined by historical transmission records and historical vector surveillance records) and has not reported any travel to a high endemic area. Details of this surveillance system employed are reported elsewhere [5, 14]. Data from each facility were used to generate maps, while the active investigation data were used to examine map accuracies.

### Ecological/environmental covariates

Long-term minimum, maximum and mean land surface temperature and precipitation data were extracted from the WorldClim datasets [15]. Enhanced Vegetation Index (EVI), Normalized Difference Vegetation Index (NDVI) and Normalized Difference Water Index (NDWI), derived from MODIS data at 250 m resolution, were extracted from Google Earth Engine [16]. As the case data span three years, mean EVI and NDVI were calculated for the month of January in the years 2011 to 2013. January was chosen as this represents the month with the highest rainfall. The Shuttle Radar Topography Mission (SRTM) digital elevation model at 90 m resolution was used to estimate altitude [17]. Topographic Wetness Index (TWI) was calculated using methods described elsewhere [18]. Land cover data were obtained using the most recent (2009) datasets generated by the GlobCover project at 300 m resolution [19]. To ensure that the land cover classes used for modelling were the same as those used for country wide prediction, the land cover classes were reclassified into six groups (1 - cultivated terrestrial areas and managed lands; 2 – woody trees; 3 – shrub; 4 – herbaceous; 5 - artificial and bare areas; 6 water bodies). Data on population density, available for the year 2010, were downloaded from the Worldpop project (http://www.worldpop.org.uk) at ~100 m resolution. Distance to nearest river was calculated with ArcGIS using Digital Chart of the World data on locations of rivers available online [20]. All covariate data were resampled to the same extent at 1 km resolution using the 'raster’ package in R [21]. Resampling of population data involved summing pixel values to preserve the same total population. Resampling of elevation, EVI, NDVI, NDWI, temperature, precipitation and distance to river layers was done using bilinear interpolation, whereas categorical land cover data were resampled using the nearest neighbour method. All continuous covariates were centered around zero and scaled by dividing by the standard deviation.

### Catchment areas

**Figure 1:**
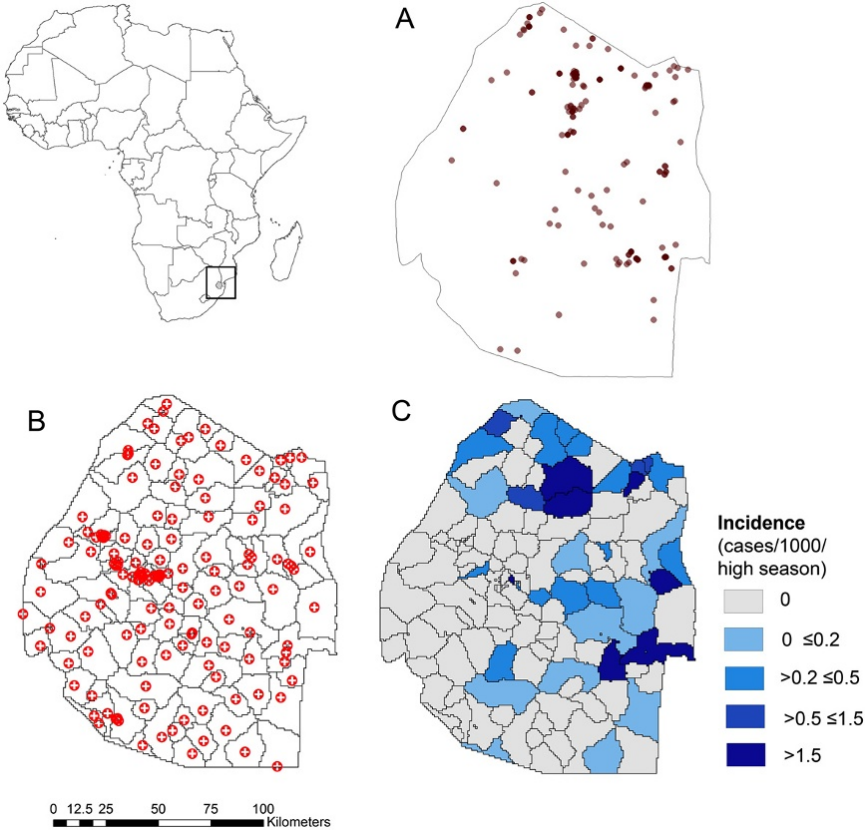
**Map of Africa showing the location of Swaziland (inset map). A** - Household location of cases occurring between January-April 2011–2013 within Swaziland, **B** – Locations of health facilities offering malaria diagnosis with modelled catchment areas based on travel time and **C** – Estimated incidence of cases per catchment area.

As health facility catchment area boundaries are not available, these were estimated using travel times generated using a travel model which accounts for slope, land cover and road and river layers to generate a travel time surface. Full details of this approach are found elsewhere [22]. Briefly, Tobler’s hiking function [23] was used to calculate walking speed as a function of slope (Table 1). The speed at which at individual can walk was also assumed to be influenced by land cover type, with rivers being impassable by foot. Assuming individuals travel by road transport when reaching a road, travel speed along roads was assigned according to road type (Table 1). Slope was calculated using the SRTM digital elevation model with land cover type calculated using GlobCover data and locations of rivers via the Digital Chart of the World data. Road data were obtained via the Open Street Map project [24] as this appeared to provide the most comprehensive data. Using satellite imagery to guide classification, Open Street Map road classes were reclassified into three groups which were assigned different travel speeds: a) motorways and trunk roads; b) primary and secondary roads; and c) tertiary and unclassified roads (Table 1). Using the gdistance package [25] in R (see Additional file 1 for example code) catchment areas were derived by identifying, for each pixel, the closest health facility offering malaria diagnosis by travel time (Figure 1B). If more than one health facility was present in a single 1 km x 1 km pixel, case data from the corresponding health facilities were merged (summed) before analysis.

**Table 1:** **Data used to estimate travel time to public health facilities in Swaziland**

Data layer	Category	Speed (km/h)
Land cover	Tree cover, broad leaved deciduous or evergreen	5
	Tree cover, needle leaved, deciduous or evergreen	5
	Tree cover, other	2
	Shrub cover	5
	Herbaceous cover	3
	Sparse herbaceous	4
	Cultivated and managed areas	5
	Bare areas/desert	2
	Water bodies/rivers	0
Digital elevation (slope)	0° (flat)	5.00
	5°	3.71
	-5°	5.27
Roads	Motorway/trunk	80
	Primary/secondary	60
	Tertiary/unclassified	10

### Treatment-seeking

As not all individuals with malaria seek treatment at public health facilities, modelling the raw case data could lead to spurious estimates of associations between risk and covariates. Such a phenomenon is analogous to the species modelling problem where certain environmental conditions, that may be related to species occurrence, influence the detectability of that species [26]. From a malaria perspective, this requires a step that accounts for the fact that not all individuals seek treatment for malaria and that this phenomenon may vary as a function of travel time to health facility. To model the relationship between treatment-seeking at a public facility and travel time to health facility, treatment-seeking data from the 2010 Swaziland MIS were used. Typically, data on where individuals with reported fever in the previous two weeks seek treatment is used to assess malaria treatment-seeking behaviour [22]. However, only 88 individuals from the Swaziland MIS reported having had fever in the previous two weeks limiting the use of this information. Instead, data from a total of 2,377 heads of households were available on the response to the question on the source, if any, of treatment sought should they, or members of their family, display signs or symptoms of malaria. Answers were recorded as 1 if the individual responded that they would visit a public facility and 0 if they would visit a private facility, traditional healer, or would not seek treatment. Using GPS coordinates for households collected during the MIS, the travel time to nearest health facility was calculated for each household using the modelled travel time surface, as described above.

### Statistical analysis

A hierarchical modelling framework [27, 28] was used to link fine scale covariate data with catchment level case data, accounting for treatment-seeking. There were 142 catchments indexed by *j* where *j* ∈ 1 : 142, and each catchment contains *n*_*j*_ pixels of 1 km^2^. Each pixel is indexed by *k* where *k* ∈ 1 : *n*_*j*_. There were also 2,377 individuals for which information on malaria treatment-seeking was available, indexed by *i* where *i* ∈ *1* : *2*, *377*.

### Cross-scale model

The relationship between travel time (*t*_*i*_) and the probability of a public facility being used (*h*_*i*_) for each individual (*i*) was modelled using a logistic regression.
1

This model was then used to predict for each pixel the probability that an individual would seek treatment for malaria in a public facility (*h*_*jk*_) for every *k*^th^ 1 km^2^ pixel in each of the *j*^*th*^ catchment in Swaziland. This was multiplied by the unobserved or 'latent’ pixel level probability of there being a case (*p*_*jk*_) to produce the pixel probability of observing a case (*d*_*jk*_), where (*p*_*jk*_) was modelled as a function of the *N* observed fine-scale environmental covariates (*X*) plus a catchment level random effect (*u*_*j*_) using logistic regression:
23

The probability of a case being observed within the *j*th catchment (*P*_*j*_) was defined as the sum of the expected number of observed cases per catchment pixel (*k* ϵ 1:*n*_*j*_) over the catchment population (*M*_*j*_), where *M*_*j*_ equals the sum of the population within each of the catchment pixels (*m*_*jk*_):
4

The number of cases per catchment *C*_*j*_ was treated as a random draw from the catchment population *M*_*j*_ with the probability of selecting a case *P*_*j*_:
5

Equations 1–5 allowed calculation of the likelihood of observing the data (*C*_*j*_) given the model and the input predictors (*t*_*i*_,*X*_*N,jk*_). The model can then be fit either by numerically maximizing the likelihood, or by Markov Chain Monte Carlo (MCMC). The latter option was chosen as it enables estimation of uncertainty about the relationships represented by *β* (Equations 1 and 3) and also the uncertainty about model predictions both at the pixel scale (*p*_*jk*_) and the catchment scale (*P*_*j*_).

To select which covariates to include in the fine-scale equation, lasso penalization, which shrinks estimates of those coefficients not associated with the outcome towards zero, was used [29]. In this way, lasso penalization acts as a way to select which covariates to retain in the model. All covariates were included in an initial lasso regression excluding a catchment level random effect. Variables with 95% Bayesian credible intervals that did not cross zero were then included in a standard (non-lasso) regression model with a catchment level random effect. Following inclusion of a catchment random effect, any variables with 95% Bayesian credible intervals that did not cross zero were retained in the final model. For model fitting, an MCMC sampler in JAGS [30] was used to run 2 MCMC chains with 50,000 iterations as burn-in and 20,000 iterations saved for inference, thinned every 20 used to store model parameter estimates. Convergence was assessed by visual inspection of trace plots of chains.

### Model evaluation

To assess model fit at the catchment scale, predicted catchment numbers of cases were estimated by multiplying the predicted probability of there being a case (posterior mean) by the population for each pixel. These values were then multiplied by the posterior mean of the predicted probability of a case presenting to a public health facility. Predicted numbers of cases per pixel were then summed across catchment areas to obtain predicted numbers of cases per health facility. These values were then rounded to the nearest whole number and compared to the observed numbers of cases at each health facility using scatter plots as well as by calculating root mean squared error (RMSE) and mean absolute error (MAE).

To evaluate the accuracy of the pixel scale predictions from the model, the fine scale predictions (posterior mean) at cases were compared to a random selection of pseudo-controls. 10,000 pseudo-controls were selected by sampling pixels with replacement from throughout Swaziland with probability proportional to the pixel population density and the probability of seeking treatment at a public health facility (using model parameters from Equation 1). Mean prediction values were compared using box-plots and Area Under the Curve (AUC), which is the probability that prediction values at a randomly selected case will be higher than at a randomly selected control. Uncertainty in the fine scale model predictions were visualized by plotting the lower (2.5%) and upper (97.5%) prediction interval of the posterior distribution of *p*_*jk*_.

## Results

Of the 812 cases that occurred between 2011–2013, 471 (58%) received a case investigation. Of those, a total of 221 locally acquired cases occurred between January and April in the years 2011 to 2013 and were included in the analysis (Figure 1A). Using modelled catchment areas to estimate catchment population (Figure 1B), catchment area incidence estimates suggested a tendency for higher risk in northern and eastern regions of the country (Figure 1C).

Of the 2,377 individuals asked about seeking treatment should they believe they had malaria, 98% answered that they would seek treatment at a public facility. Despite these high rates, results from the model showed that the odds of seeking treatment for malaria at a public facility were slightly, but significantly, negatively associated with travel time (centered and scaled) to nearest public health facility (Odds Ratio 0.99).

**Figure 2:**
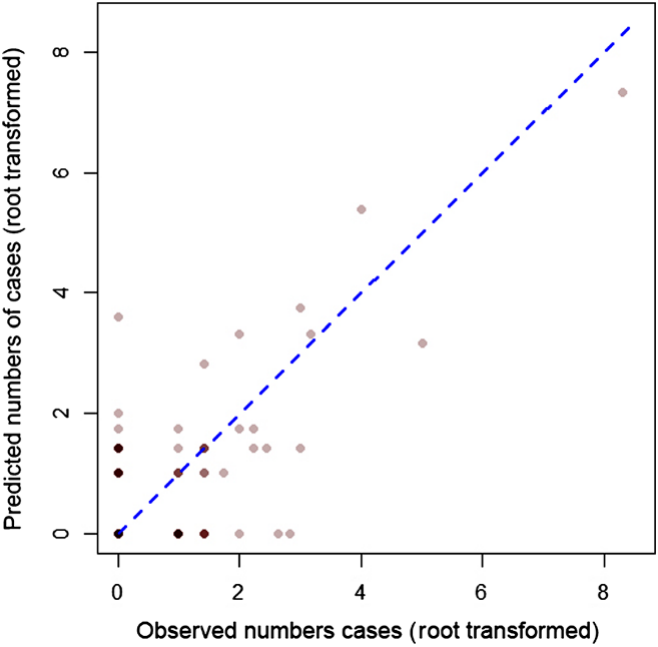
**Root transformed observed versus predicted numbers of cases per health facility.** Counts were root transformed to aid visualisation. Points are plotted with transparent colours hence darker points indicate overlapping points. The blue dashed line corresponds to a 1:1 relationship.

**Figure 3:**
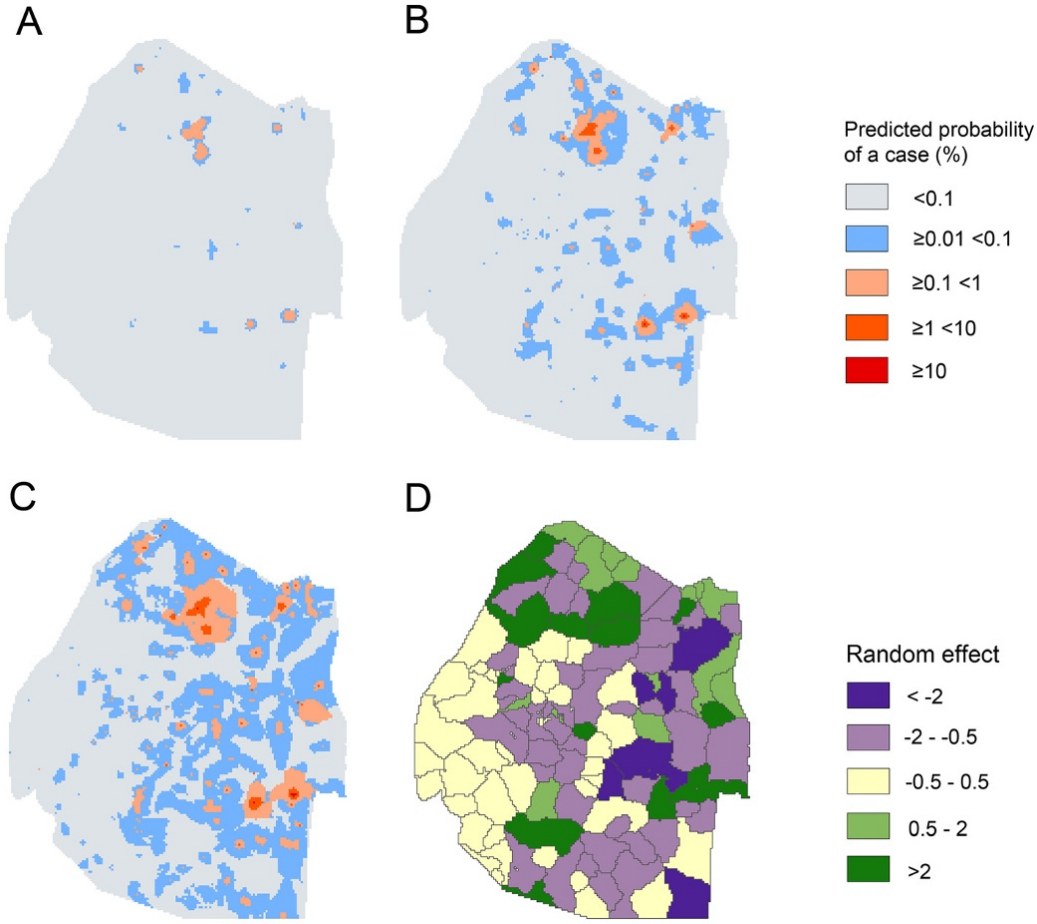
**Model predictions of the probability of a case occurring in a high season 2011–2013. A** – lower 2.5% prediction interval; **B** – mean prediction; **C** – upper 97.5% prediction interval; **D** – catchment level random effect values.

**Figure 4:**
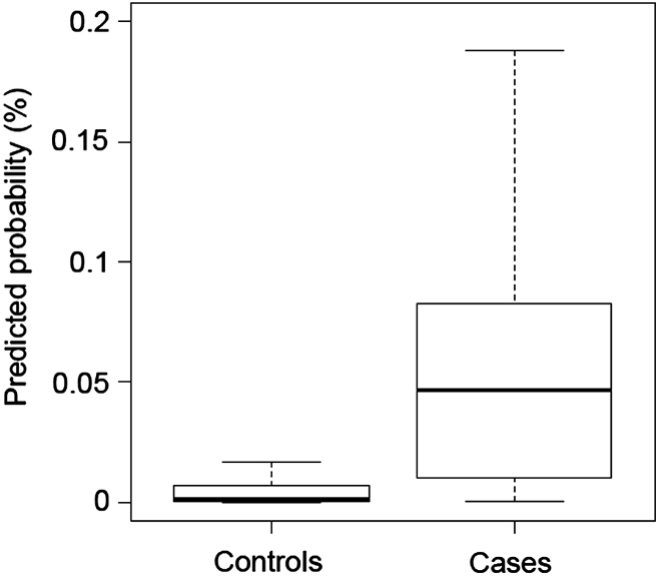
**Predicted probability (posterior mean) of a case occurring at control and case locations.** Whiskers correspond to 1.5 times the interquartile range.

In terms of pixel scale relationships between covariates and risk, following variable selection, the final model included only mean temperature which showed a positive association with probability of being a case (OR 8.15) and distance to health facility which showed a negative relationship (OR 0.12) (Table 2).When predictions were scaled up to catchment level, the predicted numbers of cases per health facility broadly corresponded with the observed numbers (Figure 2). The RMSE and the MAE of the observed versus predicted numbers of cases per health facility were 1.16 and -0.09 respectively, indicating good model fit with very little bias. 84 of the 101 (83%) health facilities with zero cases were correctly predicted to have zero cases.The mean, 2.5% and 97.5% intervals of the predicted posterior for each pixel are shown in Figure 3A-C. This shows that the pixel scale predictions broadly correspond to the coarser scale catchment level data. Figure 3D shows the value of the catchment level random effect (intercept) term mapped by catchment area. This shows that while risk in the majority of the very low risk western catchment areas was predicted correctly by land surface temperature and distance to facility alone, risk in other high and low risk areas of the country, particularly in the north-east, deviated from these predictions. Figure 4 shows the distribution of prediction values of cases versus 10,000 pseudo-controls as a box-plot. Prediction values were noticeably higher at case locations than control locations. This was reflected in the AUC value of 0.84 which indicates good discriminative capacity between cases and controls.

**Table 2:** **Model parameters estimated from the final household and cross-scale models, showing pixel scale relationships between malaria and covariates**

Variable	Mean odds ratio	BCI
Land surface temperature	8.15	3.86 – 19.31
Travel time to health facility	0.12	0.01 – 0.75

Note that odds ratios refer to centered and scaled covariates. BCI are Bayesian 95% credible intervals.

## Discussion

As malaria transmission declines, it becomes increasingly focal. In order to target resources accordingly, an understanding of transmission risk over fine scales is required. Traditionally, such risk mapping is done using cross-sectional infection prevalence surveys, such as malaria indicator surveys. In low transmission settings such surveys do not produce the number of positives required for risk mapping or decision making [4]. While a handful of malaria elimination programmes, such as Swaziland [5] and the Solomon Islands and Vanuatu [31], map the households of malaria cases facilitating fine scale risk mapping, most countries have to rely on health facility level case data. This paper describes a method that uses routine health facility malaria case data in conjunction with freely available remotely sensed data to predict malaria risk, and associated uncertainty, down to a scale of 1 km^2^.

While this example is restricted to the cross-scale prediction of malaria risk from health facility level data in Swaziland, this approach holds promise for cross-scale modelling and prediction of malaria in other transmission settings. In particular, this method is well suited to situations where prevalence is low (i.e. <3%) but the number of cases is still too high to allow follow up and mapping. The implementation of this approach in other settings requires several considerations. Firstly, in this study, the estimation of catchment boundaries was done only for those health facilities offering malaria diagnosis. In settings where this information is not known, catchment boundaries would have to be generated for each health facility, which could affect predictions. Secondly, only cases classified as local were used in the modelling process. If cases are not correctly classified according to origin of infection (i.e. if there is an under or over estimation on the proportion of cases that are imported), this approach is likely to lead to unreliable predictions. Thirdly, in Swaziland, catchment areas are reasonably small, due to the relatively small size of the country and the high population coverage of the public health system. How well this method works in settings where catchment areas are larger, and encompass a wider range of environmental conditions, is not clear. Fourthly, while treatment-seeking data are collected as part of MIS or Demographic Health Surveys, for some countries this information does not exist. In these settings, a relationship could be assumed using data from neighbouring countries or, ideally, a representative survey could be undertaken. Conducting sensitivity analyses, varying rates of treatment seeking, could also be a useful tool to assess the impact of this parameter on model predictions. This was not done here as it was beyond the scope of the study which focuses more on showing proof of concept. Fifthly, in settings with different health systems, for example where surveillance consists of community- and facility-based case detection or where the private sector plays a more prominent role, slightly different models would be required. Finally, the specific models used should be appropriate for the case data used. In this study, logistic regression was used to estimate pixel-level relationships due to the very low numbers of cases across the country. In other settings, employing alternative methods, such as zero-inflated, negative binomial or Poisson regression models, may be more appropriate.

Though not the main focus of this study, results from the model suggest that a positive relationship between temperature and risk of malaria exists at the pixel level. This fits with our understanding of the disease, as warmer temperatures are more conducive to parasite development. Unusually, the model seemed to suggest that areas further from health facilities were at reduced risk. This may be due in part to correlations between distance to health facility and other variables not included in the modelling process. For example, imported cases, proximity to which has been shown to be a risk factor for being a local case [14], could be less common in more isolated communities. Equally, population density could be much lower in more isolated communities which may reduce transmission potential.

Maps of the catchment level random effect term show that areas in the north east of the country tended to deviate the most from model predictions using temperature and distance to nearest facility. This suggests that there are other factors associated with risk in these areas that are not formally accounted for in the model. For those areas with positive random effect values (i.e. higher than expected risk given the environment) this could be due to presence of imported cases, or areas missed by IRS or ITN distribution. For those areas with negative random effect values (i.e. lower than expected risk given the environment), this could be due to high coverage of IRS or ITNs, or presence of other interventions not included in the modelling process. Equally, differences between areas could indicate differences surveillance capacity at the facility level in terms of diagnosis and reporting. Irrespective, mapping random effects is useful as it allows questions and hypotheses to be raised, other predictor variables to be considered or dubious data to be identified. Furthermore, it highlights catchment areas with no cases that may be suitable for transmission and areas unsuitable for transmission in which cases occur.

While the modelling framework has generated encouraging results with regard to cross-scale prediction, the approach outlined here has several important limitations and considerations for implementation elsewhere. Firstly, despite finding a relationship between treatment-seeking in the public sector and travel time to nearest health facility, overall rates of treatment-seeking in the public sector was extremely high at 98%. While Swaziland is a small country and does not have a large private sector, this percentage appears high. Unfortunately, the more frequently used question to assess treatment-seeking behaviour, where if at all those with a reported fever in the previous two weeks sought treatment, could not be used due to small sample sizes.

Secondly, it was assumed that individuals with malaria seek treatment at their closest health facility by travel time. While this is likely to be true in many cases, choice of health facility may be influenced by other factors such as the type and quality of service provided [32] as well as the cost of travel [33]. More complex catchment models, which include competition between different types of facilities and allow overlapping catchment areas [34], may improve the predictive accuracy of cross-scale predictive models. Equally, travel models using local data on travel preferences may improve predictions.

Thirdly, a relatively simple linear modelling approach was used to estimate the pixel scale relationships between malaria risk and covariates. There are several possible improvements which could lead to more accurate predictions. For example, generalized additive models, which would allow for more complex non-linear relationships could be explored. Similarly, including terms to account for spatial autocorrelation both at the catchment and/or pixel level might lead to more robust estimation of relationships and could help with predictions. Furthermore, a consideration of the environmental and ecological conditions at the time point at which the case occurred, or lagged as appropriate, would likely improve the accuracy of predictions and would allow time specific predictions to be made. With the availability of remotely-sensed data increasing, accessing such data should become progressively straightforward. Additionally, use of techniques such as lasso regression for covariate selection provide potential for a more automated modelling approach. While there are still a number of challenges to overcome, this raises the possibility of making these types of predictive models accessible to non-experts within malaria control programmes. These issues were not explored here due to computational limits, but are the focus of future studies.

Fourthly, it should be noted that not all cases that were diagnosed in Swaziland between 2011–2013 received an investigation, due to either resource constraints or failure to make contact with the case. The data used to build and validate these models therefore do not necessarily represent the full picture of malaria in the country. While we do not believe this introduced any bias in this case, such an issue illustrates the benefit of conducting case investigation at the health facility, which, as of 2014 is done in Swaziland. This also highlights the fact that in contrast to higher transmission settings, where predictions can be validated against gold-standard cross sectional survey data, a comparison with passively detected geolocated cases is the only method to validate predictions in this setting.

Finally, Swaziland is one of only a few programmes to have information on the household location of cases, enabling validation of the fine scale risk maps. If this information is known, there is no need for cross scale prediction and modelling can be done using the locations of case households [14]. If this information is not known, the Bayesian modelling approach described here can be used to generate estimates of uncertainty in the predictions. That said, household investigation and active surveillance should still be encouraged. High rates of active testing are generally believed to be a requirement for any elimination programme [35]. Furthermore, visiting case households provides an opportunity for additional targeted interventions such as presumptive treatment, ITN distribution or IRS and allows an assessment of household risk factors. These results do, however, suggest that programmes can obtain detailed understanding in the heterogeneity of malaria transmission without these specific data.

While this paper focusses on the prediction of fine scale malaria transmission risk from health facility data, this modelling framework has potential utility in other multi-scale modelling problems. This could be, for example, fine scale prediction of risk from district or school level disease data. Equally, this method could be used to look at the impact of fine scale interventions, such as village level ITN distributions, on catchment level malaria incidence. Similarly, this approach could be applied to modelling and predicting other environmentally and ecologically driven diseases such as *Plasmodium vivax*[36], schistosomiasis [28, 37], soil-transmitted helminths [38, 39] and lymphatic filariasis [40]. This is particularly true for low transmission settings, where large scale prevalence surveys become inefficient due to very large sample size requirements to find positives [3].

## Conclusions

As malaria transmission declines, interventions need to be deployed with increasing granularity. Often, however, the case data used to understand spatial patterns of risk is only available at the health facility or district level, limiting decision making to this resolution. Using a novel modelling framework, this study has shown that it is possible to combine health facility level case data with fine scale environmental and climatological data to predict malaria risk at fine resolution. This information can help to guide decision making at sub-catchment levels, to ensure interventions are targeted in as evidenced based way possible.

## Electronic supplementary material

Additional file 1: **R code to calculate health facility catchment areas based on travel time using the gdistance package.** (DOCX 17 KB)

## References

[1] Guerra C, Hay S, Lucioparedes L, Gikandi P, Tatem A, Noor A, Snow R (2007). Assembling a global database of malaria parasite prevalence for the Malaria Atlas Project. Malar J.

[2] Gething P, Patil A, Smith D, Guerra C, Elyazar I, Johnston G, Tatem A, Hay S (2011). A new world malaria map: *Plasmodium falciparum* endemicity in 2010. Malar J.

[3] Hay SI, Smith DL, Snow RW (2008). Measuring malaria endemicity from intense to interrupted transmission. Lancet Infect Dis.

[4] Hsiang MS, Hwang J, Kunene S, Drakeley C, Kandula D, Novotny J, Parizo J, Jensen T, Tong M, Kemere J, Dlamini S, Moonen B, Angov E, Dutta S, Ockenhouse C, Dorsey G, Greenhouse B (2012). Surveillance for malaria elimination in Swaziland: a national cross-sectional study using pooled PCR and serology. PLoS One.

[5] Sturrock HJW, Novotny JM, Kunene S, Dlamini S, Zulu Z, Cohen JM, Hsiang MS, Greenhouse B, Gosling RD (2013). Reactive case detection for malaria elimination: real-life experience from an ongoing program in Swaziland. PLoS One.

[6] Kunene S, Phillips AA, Gosling RD, Kandula D, Novotny JM (2011). A national policy for malaria elimination in Swaziland: a first for sub-Saharan Africa. Malar J.

[7] Kazembe LN (2007). Spatial modelling and risk factors of malaria incidence in northern Malawi. Acta Trop.

[8] Clements AC, Barnett AG, Cheng ZW, Snow RW, Zhou HN (2009). Space-time variation of malaria incidence in Yunnan province. China Malar J.

[9] McPherson JM, Jetz W, Rogers DJ (2006). Using coarse-grained occurrence data to predict species distributions at finer spatial resolutions—possibilities and limitations. Ecol Model.

[10] Hartley S, Kunin WE, Lennon JJ, Pocock MJO (2003). Coherence and discontinuity in the scaling of specie's distribution patterns. Proc R Soc Lond B Biol Sci.

[11] Araújo MB, Thuiller W, Williams PH, Reginster I (2005). Downscaling European species atlas distributions to a finer resolution: implications for conservation planning. Glob Ecol Biogeogr.

[12] Niamir A, Skidmore AK, Toxopeus AG, Muñoz AR, Real R (2011). Finessing atlas data for species distribution models. Divers Distrib.

[13] Keil P, Belmaker J, Wilson AM, Unitt P, Jetz W (2013). Downscaling of species distribution models: a hierarchical approach. Methods Ecol Evol.

[14] Cohen J, Dlamini S, Novotny J, Kandula D, Kunene S, Tatem A (2013). Rapid case-based mapping of seasonal malaria transmission risk for strategic elimination planning in Swaziland. Malar J.

[15] Hijmans RJ, Cameron SE, Parra JL, Jones PG, Jarvis A (2005). Very high resolution interpolated climate surfaces for global land areas. Int J Climatol.

[16] **Google Earth Engine** [https://earthengine.google.org]

[17] Jarvis A, Reuter HI, Nelson A, Guevara E (2008). Hole-Filled Seamless SRTM Data V4.

[18] Cohen JM, Ernst KC, Lindblade KA, Vulule JM, John CC, Wilson ML (2010). Local topographic wetness indices predict household malaria risk better than land-use and land-cover in the western Kenya highlands. Malar J.

[19] European Space Agency ESAGP, led by MEDIAS-France: **GlobCover Land Cover v2 2009 database.** http://due.esrin.esa.int/globcover/

[20] **DIVA-GIS** http://www.diva-gis.org

[21] R Development Core Team (2008). R: A Language and Environment for Statistical Computing.

[22] Alegana V, Wright J, Pentrina U, Noor A, Snow R, Atkinson P (2012). Spatial modelling of healthcare utilisation for treatment of fever in Namibia. Int J Health Geogr.

[23] Tobler W (1993). Three Presentations on Geographical Analysis and Modeling.

[24] **Open Street Map** http://www.openstreetmap.org

[25] Van Etten J (2012). R Package Gdistance: Distances and Routes on Geographical Grids (version 1.1-4).

[26] Kéry M (2010). Introduction to WinBUGS for Ecologists.

[27] Royle J, Dorazio R (2008). Hierarchical Modeling and Inference in Ecology: the Analysis of Data from Populations, Metapopulations and Communities.

[28] Sturrock HJW, Pullan RL, Kihara JH, Mwandawiro C, Brooker SJ (2013). The use of bivariate spatial modeling of questionnaire and parasitology data to predict the distribution of *Schistosoma haematobium* in coastal Kenya. PLoS Negl Trop Dis.

[29] Tibshirani R (1996). Regression shrinkage and selection via the lasso. J Royal Stat Soc B.

[30] Plummer M (2003). JAGS: a program for analysis of Bayesian graphical models using Gibbs sampling. Proceedings of the 3rd International Workshop on Distributed Statistical Computing (DSC 2003), March 20–22.

[31] Kelly GC, Seng CM, Donald W, Taleo G, Nausien J, Batarii W, Iata H, Tanner M, Vestergaard LS, Clements AC (2011). A spatial decision support system for guiding focal indoor residual interventions in a malaria elimination zone. Geospat Health.

[32] Noor A, Amin A, Gething P, Atkinson P, Hay S, Snow R (2006). Modelling distances travelled to government health services in Kenya. Trop Med Int Health.

[33] Hjortsberg CA, Mwikisa CN (2002). Cost of access to health services in Zambia. Health Policy Plan.

[34] Guagliardo M (2004). Spatial accessibility of primary care: concepts, methods and challenges. Int J Health Geogr.

[35] Sturrock HJW, Hsiang MS, Cohen JM, Smith DL, Greenhouse B, Bousema T, Gosling RD (2013). Targeting asymptomatic malaria infections: active surveillance in control and elimination. PLoS Med.

[36] Gething PW, Elyazar IRF, Moyes CL, Smith DL, Battle KE, Guerra CA, Patil AP, Tatem AJ, Howes RE, Myers MF, George DB, Horby P, Wertheim HF, Price RN, Müeller I, Baird JK, Hay SI (2012). A long neglected world malaria map: *Plasmodium vivax* endemicity in 2010. PLoS Negl Trop Dis.

[37] Clements AC, Firth S, Dembele R, Garba A, Toure S, Sacko M, Landoure A, Bosque-Oliva E, Barnett AG, Brooker S, Fenwick A (2009). Use of Bayesian geostatistical prediction to estimate local variations in *Schistosoma haematobium* infection in western Africa. Bull World Health Organ.

[38] Pullan RL, Gething PW, Smith JL, Mwandawiro CS, Sturrock HJW, Gitonga CW, Hay SI, Brooker S (2011). Spatial modelling of soil-transmitted helminth infections in Kenya: a disease control planning tool. PLoS Negl Trop Dis.

[39] Magalhães RJS, Clements ACA, Patil AP, Gething PW, Brooker S (2011). The applications of model-based geostatistics in helminth epidemiology and control. Adv Parasitol.

[40] Slater H, Michael E (2013). Mapping, Bayesian geostatistical analysis and spatial prediction of lymphatic filariasis prevalence in Africa. PLoS One.

