# Supplementary material for: Fine-scale malaria risk mapping from routine aggregated case data
Source: Malar J. 2014 Nov 3;13:421. doi: 10.1186/1475-2875-13-421 (PMC4349235; doi:10.1186/1475-2875-13-421)
Supplement: Supplementary file 1 — Additional file 1: R code to calculate health facility catchment areas based on travel time using the gdistance package. (DOCX 17 KB) [file 12936_2014_3687_MOESM1_ESM.docx]

**Additional file 1.** R code to calculate health facility catchment areas based on travel time using the gdistance package

library(raster)

library(gdistance)

library(maptools)

**# Read in environmental data**

SwaziElevation<-raster("") # clipped elevation raster

SwaziRivers<-readShapeSpatial("")

SwaziLandCover<-raster("")

**# Read in roads files (downloaded from OpenStreetMaps)**

Motorway<-readShapeSpatial("....osm_motorway")

TrunkRoads<-readShapeSpatial(".......osm_trunk")

PrimaryRoads<-readShapeSpatial("......osm_primary")

SecondaryRoads<-readShapeSpatial(".......osm_secondary")

TertiaryRoads<-readShapeSpatial(".......osm_tertiary")

UnclassifiedRoads<-readShapeSpatial("........osm_unclassified")

**# Generate lower resolution elevation raster**

**# This is the base layer which determines final raster resolution of other layers**

SwaziElevation<-aggregate(SwaziElevation,fact=10,fun=mean)

**# Resample Landcover raster to fit elevation raster**

SwaziLandCover<-resample(SwaziLandCover,SwaziElevation,method="ngb")

**# Build a raster of roads/rivers that overlays elevation raster**

MotorwayRaster<-rasterize(Motorway,SwaziElevation,1)

TrunkRoadsRaster<-rasterize(TrunkRoads,SwaziElevation,1)

MotorwayRaster[TrunkRoadsRaster[]==1]<-1 # combines the two

PrimaryRoadsRaster<-rasterize(PrimaryRoads,SwaziElevation,1)

SecondaryRoadsRaster<-rasterize(SecondaryRoads,SwaziElevation,1)

PrimaryRoadsRaster[SecondaryRoadsRaster[]==1]<-1 # combines the two

TertiaryRoadsRaster<-rasterize(TertiaryRoads,SwaziElevation,1)

UnclassifiedRoadsRaster<-rasterize(UnclassifiedRoads,SwaziElevation,1)

TertiaryRoadsRaster[UnclassifiedRoadsRaster[]==1]<-1 # combines the two

**# Repeat for rivers**

SwaziRiversRaster<-rasterize(SwaziRivers,SwaziElevation,1)

**# Change NA values to 0 for later analysis**

MotorwayRaster[is.na(MotorwayRaster[])]<-0

PrimaryRoadsRaster[is.na(PrimaryRoadsRaster[])]<-0

TertiaryRoadsRaster[is.na(TertiaryRoadsRaster[])]<-0

SwaziLandCover[is.na(SwaziLandCover[])]<-0

SwaziRiversRaster[is.na(SwaziRiversRaster[])]<-0

**# Import health facility coordinates**

HF_coords<-read.csv("")

**# Calculate transition across different directions from each pixel**

heightDiff <- function(x){x[2] - x[1]}

hd <- transition(SwaziElevation,heightDiff,8,symm=FALSE)

slope <- geoCorrection(hd, scl=FALSE)

**# restrict estimates of speed to adjacent cells**

adj <- adjacent(SwaziElevation, cells=1:ncell(SwaziElevation), pairs=TRUE, directions=8)

**# Use Tobler's hiking function to define speed (km/h) as a function of slope**

speed <- slope

speed[adj] <- 6*exp(-3.5 * abs(slope[adj] + 0.05))

**# Adjust according to GlobCover (see Table 1 for speeds)**

WaterAdj<-adj[SwaziLandCover[adj[,2]]==210,] # waterbodies

TreeOther<-adj[SwaziLandCover[adj[,2]]>=100 & SwaziLandCover[adj[,2]]<=120,] # Other tree cover

Herbaceous<-adj[SwaziLandCover[adj[,2]]==140,] # Herbaceous

SparseHerbaceous<-adj[SwaziLandCover[adj[,2]]==150,] # Sparse herbaceous

BareAreas<-adj[SwaziLandCover[adj[,2]]==200,] # Sparse herbaceous

speed[WaterAdj]<-0

speed[TreeOther]<-speed[TreeOther]*0.4 # (i.e. 2 km/h on flat (2/5ths of normal 5 km/h))

speed[Herbaceous]<-speed[Herbaceous]*0.6

speed[SparseHerbaceous]<-speed[SparseHerbaceous]*0.8

speed[BareAreas]<-speed[BareAreas]*0.4

**# Block movement acros major rivers (before adding roads)**

RiversAdj<-adj[SwaziRiversRaster[adj[,2]]==1,]

speed[RiversAdj]<-0

**# Add speed of roads.**

**# Restrict to adjacent 'road' cells. If going to a road cell then assign as road movement, coming from**

**# road to off road assign as walk**

TertiaryRoadAdj<-adj[TertiaryRoadsRaster[adj[,2]]==1,]

speed[TertiaryRoadAdj]<-10 # assumes 10km/h for roads

PrimaryRoadAdj<-adj[PrimaryRoadsRaster[adj[,2]]==1,]

speed[PrimaryRoadAdj]<-60 # assumes 60km/h for roads

MotorwayAdj<-adj[MotorwayRaster[adj[,2]]==1,]

speed[MotorwayAdj]<-80 # assumes 80km/h for roads

**# Now geocorrect speed to get conductance values (required for next step)**

x <- geoCorrection(speed, scl=FALSE)

**# Calculate catchment based on minimum cost distance**

**# Extract the coordinates for the raster**

RasterCoords<-coordinates(SwaziElevation)

DistToHF<-costDistance(x,RasterCoords[-which(is.na(SwaziElevation[])),],as.matrix(HF_coords))

NearestHF<-apply(DistToHF, 1, which.min)

**# Visualise results**

NearestHF_raster<-SwaziElevation

NearestHF_raster[which(!is.na(SwaziElevation[]))]<-NearestHF

plot(NearestHF_raster)
